# Raman spectroscopy and convolutional neural networks for monitoring biochemical radiation response in breast tumour xenografts

**DOI:** 10.1038/s41598-023-28479-2

**Published:** 2023-01-27

**Authors:** Alejandra M. Fuentes, Apurva Narayan, Kirsty Milligan, Julian J. Lum, Alex G. Brolo, Jeffrey L. Andrews, Andrew Jirasek

**Affiliations:** 1 Department of Physics, The University of British Columbia Okanagan Campus, Kelowna, Canada; 2 Department of Computer Science, Western University, London, Canada; 3 Department of Computer Science, The University of British Columbia Okanagan Campus, Kelowna, Canada; 4 Department of Biochemistry and Microbiology, The University of Victoria, Victoria, Canada; 5 Department of Chemistry, The University of Victoria, Victoria, Canada; 6 Department of Statistics, The University of British Columbia Okanagan Campus, Kelowna, Canada

**Keywords:** Raman spectroscopy, Machine learning, Cancer therapy, Cancer, Mathematics and computing

## Abstract

Tumour cells exhibit altered metabolic pathways that lead to radiation resistance and disease progression. Raman spectroscopy (RS) is a label-free optical modality that can monitor post-irradiation biomolecular signatures in tumour cells and tissues. Convolutional Neural Networks (CNN) perform automated feature extraction directly from data, with classification accuracy exceeding that of traditional machine learning, in cases where data is abundant and feature extraction is challenging. We are interested in developing a CNN-based predictive model to characterize clinical tumour response to radiation therapy based on their degree of radiosensitivity or radioresistance. In this work, a CNN architecture is built for identifying post-irradiation spectral changes in Raman spectra of tumour tissue. The model was trained to classify irradiated versus non-irradiated tissue using Raman spectra of breast tumour xenografts. The CNN effectively classified the tissue spectra, with accuracies exceeding 92.1% for data collected 3 days post-irradiation, and 85.0% at day 1 post-irradiation. Furthermore, the CNN was evaluated using a leave-one-out- (mouse, section or Raman map) validation approach to investigate its generalization to new test subjects. The CNN retained good predictive accuracy (average accuracies 83.7%, 91.4%, and 92.7%, respectively) when little to no information for a specific subject was given during training. Finally, the classification performance of the CNN was compared to that of a previously developed model based on group and basis restricted non-negative matrix factorization and random forest (GBR-NMF-RF) classification. We found that CNN yielded higher classification accuracy, sensitivity, and specificity in mice assessed 3 days post-irradiation, as compared with the GBR-NMF-RF approach. Overall, the CNN can detect biochemical spectral changes in tumour tissue at an early time point following irradiation, without the need for previous manual feature extraction. This study lays the foundation for developing a predictive framework for patient radiation response monitoring.

## Introduction

Breast cancer is the most common malignancy among Canadian women, accounting for 25% of all new cancer cases and 13% of all female cancer deaths^1^. Surgical resection constitutes the primary treatment for early breast cancer, achieving tumour control rates of 50–70%^2^. Furthermore, post-operative radiotherapy prescribed to the breast and lymph nodes has shown to be an effective tool in the management of breast cancer, leading to increased disease control and survival with good cosmetic results^3,4^.

Radiotherapy (RT) utilizes high energy ionizing radiation to destroy tumour tissue, while minimizing damage to the healthy surrounding tissues. Due to its effectiveness, RT is prescribed to over 50% of all cancer patients as part of their treatment^2,5^. Nevertheless, a proportion of breast cancer patients do not respond positively to radiotherapy, leading to recurrence rates as high as 42%^3^. The extent of tumour response to radiotherapy may be partially attributed to intrinsic factors, including altered metabolic pathways in cancer cells and their surrounding environment, leading to radiation resistance and disease progression^6–9^. Therefore, a tool that detects and monitors changes in metabolic signatures of radioresistance can potentially assist with identifying individuals with resistant tumours early during treatment, leading to personalized treatment strategies (e.g., dose escalation or radiosensitizing drugs) for such individuals.

Raman spectroscopy (RS) is a non-destructive, label-free optical spectroscopic modality that can monitor post-irradiation biomolecular changes in tumour cells and tissues^10–12^, offering the potential for evaluating a patient’s response to treatment^13^.

The Raman spectra of biological samples consist of several complex peaks that capture information of multiple biomolecules simultaneously. It is a challenging task to manually extract discriminative features from large data sets to develop predictive models that can guide clinical decisions. For that reason, Raman spectroscopy is often paired with machine learning techniques to facilitate spectral analysis^14,15^.

Matthews et al. have successfully applied Raman spectroscopy and principal component analysis (PCA) to characterize radiation-induced biochemical changes in individual cancer cells and tumour tissue^16^. Their work found radiation-induced increase of intracellular glycogen in H460 (lung) and MCF-7 (breast) cell lines^16^ and non-small cell lung cancer xenografts^12^. This signature was correlated with increased radiation resistance. An alternative approach, called group and basis restricted non-negative matrix factorization and random forest (GBR-NMF-RF), has also shown promise for simultaneous monitoring of multiple biochemicals in irradiated cells^17,18^. Briefly, this algorithm decomposes the Raman spectra into a weighted combination of bases spectra of constrained biochemical bases with their corresponding scores. The bases are selected by the user from a library containing Raman spectra of pure cellular biomolecules. Hence, the scores can be used to monitor specific radiation-induced molecular responses in tumour cells and tissues^14^.

Convolutional Neural Networks (CNN) are a state-of-the art deep learning algorithm designed for computer vision. Their architecture is inspired by patterns of the mammalian visual cortex, where cells are sensitive to small sub-regions of the visual field^19–21^. CNNs perform automated feature extraction directly from data by operating the input through several layers of convolution filters, each of which captures different representations of the data. The values of the convolution filters are determined during model training in a supervised manner. The generated features can then be employed for specific tasks, such as classification. This end-to-end learning approach eliminates the need for manual feature extraction (i.e., dimensionality reduction), exceeding the classification efficiency of other machine learning methods^22^. Furthermore, CNNs consider the spatial correlation of elements of the input data by enforcing a local connectivity pattern between neurons of adjacent layers, called receptive fields. This trait makes CNNs suitable for image and signal analysis.

In recent years, there has been a growing interest in using CNN-based architectures for medical image analysis to assist with accurate and rapid detection of various pathologies^23^. Furthermore, a number of publications have combined CNN models with Raman spectra of biological samples for disease screening and diagnosis, including tongue squamous cell carcinoma^24^, prostate cancer bone metastasis^25^, pancreatic^26^ and breast cancer^27^. The authors reported effective discrimination between healthy and malignant samples with superior accuracy compared to common machine learning techniques, including linear discriminant analysis (LDA) and support vector machines (SVMs). In summary, current research shows that CNN combined with Raman spectroscopy offer great potential for automated and accurate discrimination of clinical tumour tissue types. Thus, we are here interested in CNNs as a predictive model to characterize clinical tumour response to radiation therapy, specifically, to stratify samples based on their degree of sensitivity or resistance to treatment.

In this work, a CNN is built and trained for identifying post-irradiation biochemical spectral changes in breast tumour xenografts. The classification performance of the CNN is compared to that of the GBR-NMF-RF model, and we find that CNN offers improved discrimination between Raman spectra of irradiated and nonirradiated MDA-MB-231 tumours. Finally, the GBR-NMF decomposition reveals specific contributions of amino acids and lipids to the radiation response of this breast cell line.

## Methods

### Mouse model

The Raman spectra used in this study were collected from a previously developed mouse model in our lab^28^. All animal procedures were approved by the University of Victoria Animal Care Committee (Victoria, BC) and were performed following the guidelines set by the Canadian Council on Animal Care. All animal methods and results are reported in accordance with the ARRIVE guidelines. NOD.CB17-Prkdcscid/J female mice were obtained from British Columbia Cancer Research Center (BCCRC) Animal Resource Center (Vancouver, BC). Mice were housed in microisolator cages and given access to food and water ad libitum. Mice were allowed one week to adjust to the environment before starting the experiments^28^.

The human breast cancer cell line MDA-MB-231 was purchased from American Type Culture Collection (Manassas, VA, USA). Cells were cultured and injected subcutaneously into the right flank of each mouse at a concentration of 5 × 10^6^ cells in 0.1 ml phosphate buffered saline (PBS)^28^.

### Tumour irradiation and harvesting

Mice were randomized into treatment groups once their tumour size reached a predetermined end point. Animals were anesthetized via isoflurane inhalation (1-3%, in oxygen) and exposed to single fractions of 0, 5 or 15 Gy produced by a small animal irradiator (Gulmay Medical Inc., Suwanee, GA) using two 220 kVp parallel opposed fields delivered to the tumour at a dose rate of 4 Gy/min. Following 1- or 3-days post-irradiation, animals were euthanized using isoflurane overdose (5%, in oxygen) followed by cervical dislocation, and tumours were removed. Embedded tumours were snap-frozen in liquid nitrogen and stored at – 80 $^\circ$C. For each mouse, three consecutive tumour slices were prepared using a rotary microtome (MICROM International GmbH, Walldorf, Germany) and placed on magnesium fluoride slides for Raman spectroscopy^28^.

In this study, a total of 13 mice were assessed 3 days following irradiation (4 mice each given 0 and 5 Gy, and 5 mice given 15 Gy), and 6 mice were assessed 1 day following irradiation (3 mice each given 0 and 15 Gy)^28^.

### Raman spectroscopic acquisition and spectral processing

Raman spectral maps were collected using a Renishaw inVia Raman microscope (Renishaw Inc, Illinois, USA) operating with a 785 nm excitation laser with sampling volume 2 × 5 × 10 µm^3^ and power density 0.5 mW/µm^3^, a 100× dry objective (NA = 0.9) and a charge-coupled device (CCD) detector. For each tumour section, two maps were acquired from randomly selected regions covering an area of 100 × 100 µm^2^ or 200 × 200 µm^2^, with step size 15 µm and 20 s collection time per point^28^.

Each spectrum was pre-processed to remove cosmic rays, reduce noise via spectral smoothing, subtract background arising from substrate and biological fluorescence, correct for wavenumber calibration drifts, and normalize to the total area under the curve, as in previous studies^12,28,29^. All pre-processing was performed using previously written MATLAB algorithms (version R2014B, MathWorks Inc, MA, USA).

The final data set consisted of 3054 spectra acquired at day 1 and 6708 spectra acquired at day 3 post-irradiation.

### CNN model building and architecture

**Figure 1:**
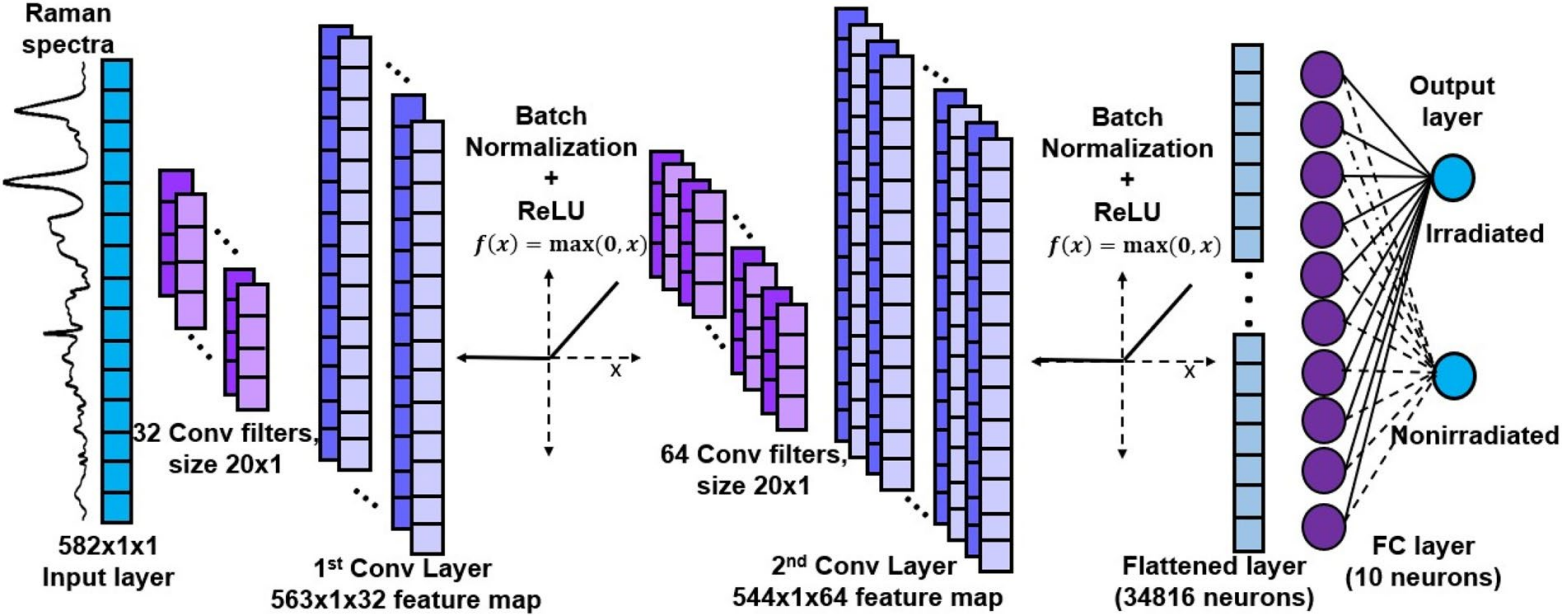
CNN architecture for Raman spectroscopy analysis.

A one-dimensional CNN for Raman spectra classification was developed in MATLAB (version R2021a) using the Deep Learning Toolbox. The CNN architecture and parameter optimization was performed with the data acquired at day 3 post-irradiation using a trial-and-error approach. Different combinations of number of layers, convolution filter size and number, activation functions, optimization algorithm, learning rate, and regularization techniques were tested. The final one-dimensional CNN architecture used in this work is shown in Fig. 1.

The spectra are input as a 582 × 1 vector containing the intensity values sampled at regular wavenumber intervals. Two convolutional layers are located behind the input layer to perform feature extraction on the Raman spectra. With an increase in convolutional layers, the model improves representation capabilities, as more features are captured from the data^20^. However, increasing the depth of the model can lead to overfitting in smaller data sets. A CNN with two convolutional layers is consistent with models used in previous studies for spectral analysis^24,25^. The first convolutional layer contains 32 convolution filters of size 20 × 1 and stride 1. Mathematically, the convolution operation is given by^24^:

1 $$y^{j} = f(b^{j} + \sum\nolimits_{i} {k^{ij} * x^{i} } ),$$

where *y *^*j*^ is the j-th output feature map obtained from the operation, *x*^*i*^ is the i-th input map, *k*^*i j*^ is the convolution filter between *x*^*i*^ and *y *^*j*^, ∗ denotes convolution, *b *^*j*^ is the bias term, and *f* is the activation function. The size (height × width) of the convolution output, *O*, is given by^30^:

2 $$O = \frac{{({\text{I}} - K + {2}P)}}{S} + {1,}$$

where *I* refers to the input size, *K* is the size of the convolution filters, *S* is the stride, and *P* is the amount of padding, which for our CNN, *P* = 0. Finally, the number of output maps is equal to the number of filters in the layer. The output of the first convolution layer is a 563 × 1 × 32 feature map, containing the spectral features learnt from the data. The resulting map is input to the second convolutional layer, containing 64 filters of size 20 × 1 and stride 1 to extract higher order features. The result from the second convolution operation is a 544 × 1 × 64 feature map.

Batch normalization (BN) is performed after each convolution layer to standardize the output maps of a training mini-batch to subsequent layers. This operation improves the training speed and reduces overfitting^22,31^. Following each BN layer, the rectified linear unit (ReLU) activation function is applied to the convolution feature maps to add nonlinear modelling ability to the neural network^32^. The activated features are carried forward into the next layer.

The final output map is fed into fully connected layers to learn non-linear combinations of the extracted features. A dropout layer with inactivation probability of 0.1 is applied after the first fully connected layer to reduce overfitting by temporarily removing randomly selected neurons^22,33^. Finally, the output layer takes the features learnt by the model to calculate the input’s classification scores for each possible category. In the current architecture, the output layer contains two neurons to represent the irradiated and nonirradiated labels. The softmax activation function is used to transform the classification scores into values between zero and 1^21,22^, representing the probability of the input spectra belonging to a given class. The category with highest probability is selected as the model prediction.

The optimal values of the neural connection weights and convolution filters are determined during the model training in a supervised manner^19^. Briefly, the weights are randomly initialized to evaluate a set of labeled training examples. The model predictions are compared with the true values by means of a cost or error function. In our model, the cross-entropy loss function is used to determine the difference between true and predicted distributions^34^. Then, the loss function is minimized in an iterative process by gradually updating the weights with each step until the loss function converges to a minimum. For each training iteration, a subset of the training set called a mini-batch is used to evaluate the loss function and update the weights.

In this work, the Adam algorithm^35^, an extension of gradient descent, was used to optimize the network’s weights as used by others^22,27^. The learning rate was set to 0.0001, and a mini-batch size of 175 was used. Table 1 shows the set of hyperparameters chosen for the final CNN architecture.

**Table 1:** CNN hyperparameters and training details.

Implementation details	Parameter name	Selected value
CNN architecture	Number of convolution blocks	2
	Number of filters	32 (first block), 64 (second block)
	Filter size	20 × 1
Training	Mini-batch size	175
	Dropout rate	0.10
Optimization	Optimizer	Adam
	Learning rate	0.0001
	*β*_1_	0.9
	*β*_2_	0.999
	*ε*	1 × 10^−8^
	L2 regularization	0.0001
	Loss function	Cross entropy loss

### CNN model training

The CNN was trained for classifying Raman spectra of irradiated and nonirradiated breast cancer xenografts. To evaluate the model’s capability of detecting post-radiation biochemical spectral changes at different doses and timepoints, the entire Raman data were split into experimental groups according to dose and collection timepoint as shown in Table 2: spectra from mice exposed to (a) 0 or 15 Gy and sacrificed 3 days post-irradiation, (b) 0 or 5 Gy and assessed 3 days post-irradiation, and (c) 0 or 15 Gy and assessed 1 day post-irradiation.

**Table 2:** Data set groups for CNN training, testing and validation.

Experimental group	Control (0 Gy)	Irradiated	Total number of spectra	
0 Gy and 15 Gy at Day 3	2,200	2,505	4,705	
0 Gy and 5 Gy at Day 3	2,200	2,003	4,203	
0 Gy and 15 Gy at Day 1	1,514	1,540	3,054	

For each experimental group, the Raman spectra were randomly split into training, testing, and validation sets with a ratio of 70/20/10, respectively. The training set was used to train the CNN, that is, to optimize the model’s weights. The validation set was used to monitor the CNN training progress every few iterations and ensure that the model does not overfit to the training set. Finally, the testing set was used to evaluate the classification performance of the final model.

The testing accuracy, sensitivity, specificity, and F1-score metrics were calculated to assess the CNN performance. The definitions for these quantities are defined as in Ref.^36^. TP is the number of spectra correctly identified as irradiated, FP is the number of spectra incorrectly identified as irradiated, TN is the number correctly identified as nonirradiated, and FN is the number incorrectly identified as nonirradiated.

For each experimental group, the results were presented as the mean ± one standard deviation of ten runs made with different partitions of the data subsets and weights initialization using the ‘random number generator’ function in MATLAB.

To investigate the CNN generalization ability to unseen subjects, the model was trained and tested using a subject-wise^37^ or leave-one-mouse/tissue section/Raman map-out validation approach. The analysis was conducted on the day 3, 0 and 15 Gy treatment group data, which consists of spectra from 5 irradiated mice, corresponding to 15 irradiated tumour sections and 30 Raman maps.

**Figure 2:**
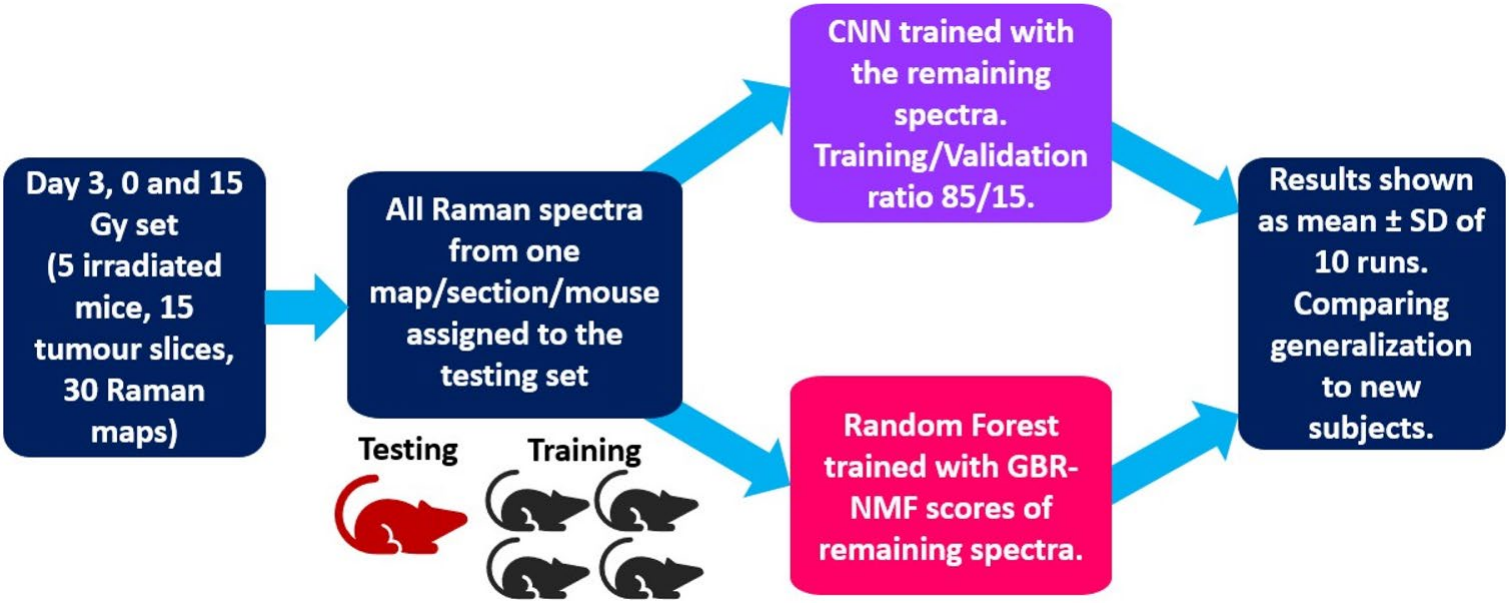
Subject-wise validation training approach.

The subject-wise validation workflow is described in Figure 2. First, the entire Raman data for a given mouse was held out of the data set. Then, the CNN was trained with the remaining spectra using an 85/15 training/validation ratio. Finally, the model was tested with the spectra of the held out mouse. The process was repeated for each of the irradiated mice in the data set, and then implemented to assess each of the corresponding tumour sections and Raman maps. The results were presented as the percentage of correctly classified Raman spectra for each mice/tumour slice/map.

### Non-negative matrix factorization and Random Forest

To visualize specific radiation-induced biochemical changes in the breast tumours with respect to dose and time, the GBR- NMF-RF model was used to obtain scores of constrained chemical bases. The GBR-NMF technique was implemented in R (version x64 4.0.3) using the algorithm developed by Shreeves et al.^17^. For all analyses, the bases matrix was constrained as a set of 31 Raman spectra of pure biochemicals (listed in Supplementary Table S1), and one unconstrained factor to represent all the other biochemical changes unspecified in the bases. Random Forest was used as a classifier to distinguish irradiated versus nonirradiated tissue spectra based on their GBR-NMF scores. Random Forest was performed using the randomForest package in R as in our previous work^14^. In addition, the Mean Decrease Accuracy (MDA) function was used to quantify the relative importance of the features (i.e., chemical bases) in the RF classification^14,18^. The number of trees forming the RF was set to 2000, and the number of variables used in each decision tree split was set to 5, and the model was trained using a 75/25 training/testing ratio or following the subject-wise validation approach with no validation set. Results of the GBR-NMF-RF model were presented as average of 10 runs.

The CNN classification performance was compared with that of the GBR-NMF-RF model. The accuracy results for both models were compared using a Wilcoxon test (p < 0.05) to determine statistically significant differences.

## Results

### Radiation response profiles in tumour tissue

**Figure 3:**
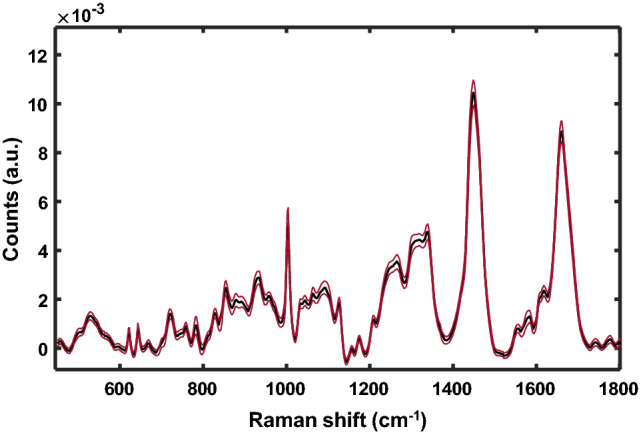
Mean Raman spectrum (black) of breast cancer xenografts exposed to 0, 5 and 15 Gy and acquired at day 3 post-irradiation. The standard deviation at each wavenumber is shown in red.

Figure 3 displays the mean Raman spectrum (black) with ± standard deviation (red) for all data collected at day 3 post-irradiation.

The greatest standard deviation appears at 1439 cm^−1^, which can be attributed to changes in lipids (CH_2_ deformation)^28,38^. Other prominent bands include those attributed to phenylalanine (1004 cm^−1^), lipids (1296, 1424, 1455, 1658 cm^−1^), tryptophan (728, 1337 cm^−1^), nucleic acids (783, 1577 cm^−1^), and lactic acid (922 cm^−1^)^39^. There are very minor differences between the mean spectrum at days 1 and 3 post-irradiation, therefore day 1 is shown in Supplementary Fig. S1.

Post-irradiation differences in the intensity of prominent Raman peaks were observed in the breast tissue spectra at both 5 and 15 Gy radiation doses. These differences correspond to changes in the biomolecular content of the tumour tissue following radiation exposure. The most prominent changes include increased lipid (720, 1063, 1126, 1448, 1658 cm^−1^), collagen (851, 928, 1448, 1658 cm^−1^)^40,41^, phenylalanine (620, 1004 cm^−1^), and tyrosine (827 cm^−1^) content, and decreased nucleic acid (790, 812 cm^−1^) bands post-irradiation^41^.

**Figure 4:**
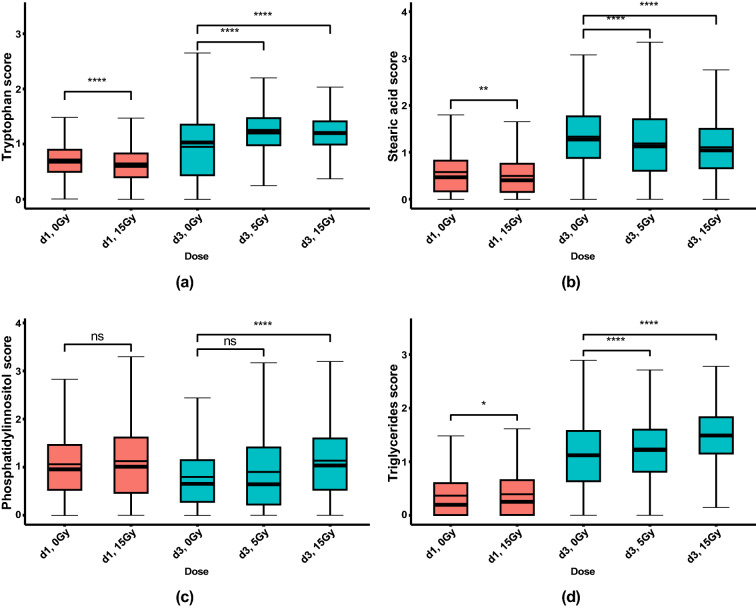
Box plots showing mean (thick line) and median (thin line) (**a**) Tryptophan, (**b**) Stearic acid, (**c**) Phosphatidylinnositol, (**d**) Triglycerides GBR-NMF scores for breast tumour xenografts exposed to 0, 5, and 15 Gy and harvested 1 and 3 days post-irradiation. Significance stars represent statistically significant score changes according to Wilcoxon’s unpaired test (*p < 0.05, **p < 0.01, ****p < 0.0001, ns = not significant).

**Figure 5:**
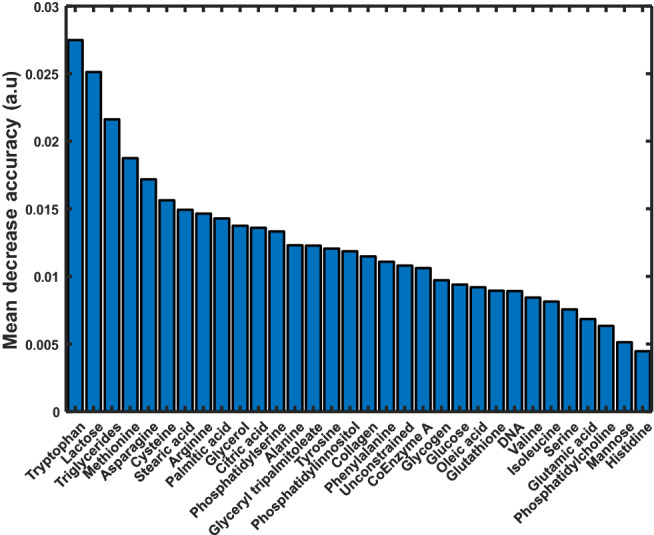
Variable importance plots of the classification of irradiated versus nonirradiated breast tissue Raman spectra based on GBR-NMF scores with Random Forest.

Figure 4 shows the mean GBR-NMF scores of four of the highest ranked chemical bases over dose level and time post-irradiation. Figure 5 displays the MDA plots of the RF classification. Different types of lipids including triglycerides, fatty acids (stearic acid), and phospholipids (phosphatidylinnositol), lactose, and amino acids were ranked as highly contributing to the observed response. These results show that the GBR-NMF-RF technique can track post-irradiation changes in multiple biochemicals throughout various post-treatment time points.

### CNN training and testing: random splitting of data set

To evaluate the CNN’s ability to detect early post-radiation spectral changes, the model was trained and tested for the classification of irradiated and non-irradiated breast tumour xenografts. In this initial evaluation, the model was trained using randomly defined training, testing, and validation sets.

**Figure 6:**
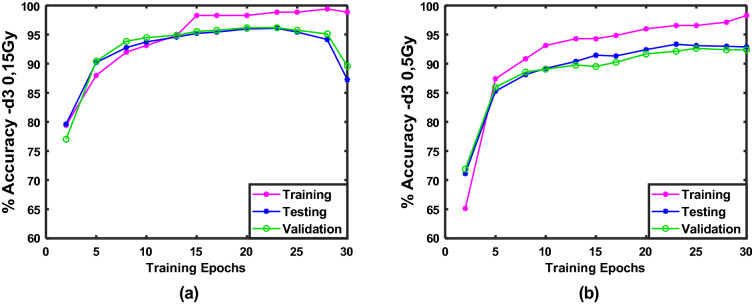
Training (magenta), testing (blue), and validation (green) accuracies plotted against number of training epochs for one run of the CNN model on (**a**) Day 3, 0 and 15 Gy and (**b**) Day 3, 0 and 5 Gy treatment groups.

Figure 6 shows the training, testing, and validation accuracy plotted against the number of training epochs for one run of the CNN on (**a**) Day 3, 0 and 15 Gy and (**b**) Day 3, 0 and 5 Gy data subsets, respectively. Similar plots were obtained for all runs on these subsets and for the Day 1, 0 and 15 Gy group. The training progress plots show, as expected, that the training accuracy improves with an increasing number of epochs, demonstrating that the CNN training is effective and stable. The validation and testing accuracies follow the same trend until reaching a point where they stop improving or decrease with increasing epochs. In order to avoid overfitting to the training set, the CNN training is stopped once the validation accuracy stops improving. The best performance epoch model is selected for the final results.

**Figure 7:**
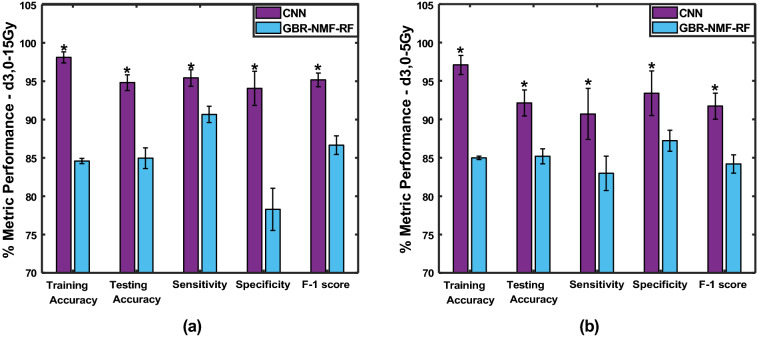
Average training accuracy, testing accuracy, sensitivity, specificity, and F-1 scores for the CNN (violet) and GBR-NMF-RF (blue) models corresponding to (**a**) Day 3, 0 and 15 Gy and (**b**) Day 3, 0 and 5 Gy treatment groups. *p<0.05.

Figure 7 shows the classification results of the CNN and GBR-NMF-RF models corresponding to the (a) Day 3, 0 and 15 Gy and (b) Day 3, 0 and 5 Gy data subsets. For both dose levels, the CNN achieved significantly higher testing accuracy, sensitivity, specificity, and F-1 score than GBR-NMF-RF in the classification of irradiated and non-irradiated tissue spectra. Specifically, the CNN distinguished nonirradiated and irradiated tissue spectra exposed to 15 Gy with testing accuracy 94.6%, while the GBR-NMF-RF model obtained a testing accuracy of 84.9%. Similarly, the CNN achieved a testing accuracy of 92.1% in the classification of nonirradiated and irradiated tissue spectra given 5 Gy, whereas the GBR-NMF-RF achieved 85.1%. These results show that the CNN is capable of accurately detecting biochemical spectral changes in breast tumour tissue at an early time point following irradiation without the need for previous manual feature extraction.

**Figure 8:**
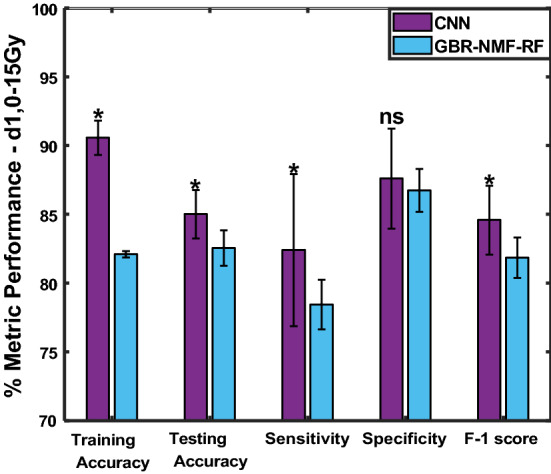
Average training accuracy, testing accuracy, sensitivity, specificity, and F-1 scores for the CNN (violet) and GBR-NMF-RF (blue) models corresponding to the Day 1, 0 and 15 Gy treatment group. *p < 0.05, ns = not significant.

Figure 8 displays the results corresponding to the Day 1, 0 and 15 Gy subset. For both models, the classification metrics were lower than those obtained for Raman spectra acquired 3 days post-irradiation. Furthermore, the CNN achieved only slightly better testing accuracy, sensitivity and F-1 score than the GBR-NMF-RF model. Specifically, the CNN obtained a testing accuracy of 85.0%, while the GBR-NMF-RF model achieved 82.5%. This could be due to the day 1 time point being too early for strong spectral changes to be detected, as seen in previous work with breast tumour cells^11,16^.

### CNN training and testing: subject-wise validation

In order for a trained predictive model to be useful in the clinical setting, it must be able to generalize to new patients/test subjects—that is, when little to no data from a given individual subject has been used to train the model. To test the generalization capability of the CNN, a subject-wise or leave-one-mouse (section and map)-out validation approach was implemented using the irradiated mice from the Day 3, 0 and 15 Gy subset.

**Figure 9:**
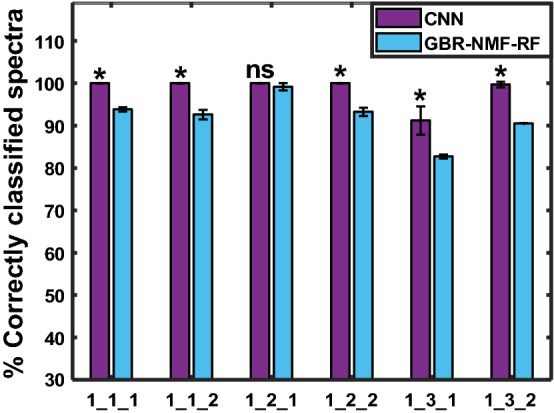
Percentage of correctly classified spectra (test accuracy) corresponding to each Raman map of mouse 1 being removed from the training set (leave-one-map-out validation). CNN (violet) and GBR-NMF-RF (blue). Map labels given as mouse number_(section #)_(map #). *Represent significant difference between CNN and GBR-NMF-RF (p < 0.05), ns = not significant.

Figure 9 shows the percentage of correctly classified spectra (i.e, test accuracy) corresponding to each of the Raman maps of irradiated mouse 1 being the test set, for both models. As seen in the figure, the CNN achieved significantly higher testing accuracy than GBR-NMF-RF for 5 out of 6 test maps. Similarly, for the rest of the mice (shown in Supplementary Fig. S2), the CNN classification performance was better than GBR-NMF-RF in the majority of cases, specifically, 19 out of 30 Raman maps. The average testing accuracy over all test Raman maps was 92*.*7 ± 4*.*6% for the CNN, and 81*.*6 ± 16*.*6% for GBR-NMF-RF.

In all leave-one-map-out validations, there was little variability among the resulting training accuracy for CNN and GBR-NMF-RF and CNN validation accuracy, with overall average values being 98*.*3 ± 0*.*2%, 85*.*4 ± 0*.*2%, 94*.*2 ± 0*.*2%, respectively. A representative result, corresponding to the maps of mouse 1, is shown in Supplementary Fig. S3.

**Figure 10:**
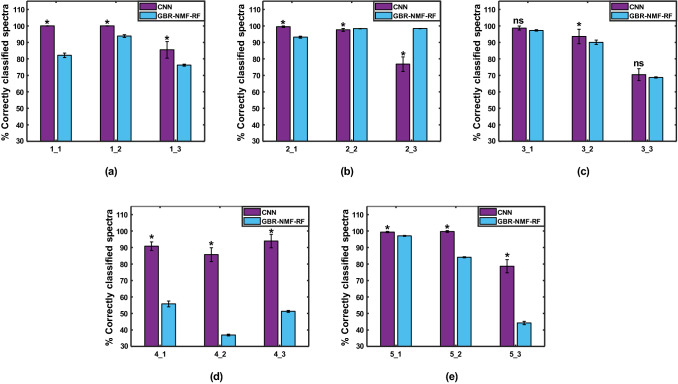
Percentage of correctly classified spectra (test accuracy) corresponding to each tumour section removed from the training set (leave-one-section-out validation). Section labels given as mouse number_(section #). *p < 0.05, ns = not significant.

Figure 10 shows the test accuracy results of the leave-one-section-out validation approach for all mouse sections. The training accuracy for CNN and GBR-NMF-RF, and CNN validation accuracy corresponding to each run are shown in Supplementary Fig. S4. In agreement with the leave-one-map-out validation, the CNN achieved a significantly higher percentage of correctly classified spectra than GBR-NMF-RF for the majority of test tissue sections (11 of 15), except for two of mice 2 and 3. The average testing accuracy over all sections was 91*.*4 ± 2*.*8% for the CNN, and 77*.*8 ± 18*.*4% for the GBR-NMF-RF. Overall, the classification improvement of CNN over GBR-NMF-RF was maintained when going from map to section-wise validation, that is, when feeding less information of a given mouse to the model training.

**Figure 11:**
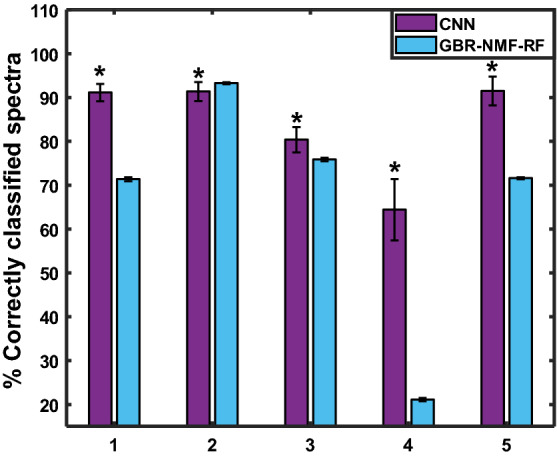
Percentage of correctly classified spectra (test accuracy) corresponding to each mouse being removed from the training set (leave-one-mouse-out validation). Section labels refer to mouse number left out of training set.

Finally, Fig. 11 shows the classification results for the leave-one-mouse-out approach. When all the spectra for the selected test mouse were removed from the training and validation sets, the percentage of correctly classified spectra per test mouse decreased in both models, compared to when only a single map or section was removed from training. This could be attributed to inter-mouse variability in the spectral signatures or intensity of radiation response. Nevertheless, in agreement with the results presented above, the CNN achieved significantly higher classification accuracy than the GBR-NMF-RF model for all except one mouse, with overall average testing accuracies of 83*.*7 ± 11*.*8% versus 66*.*6 ± 26*.*9%, respectively.

Together, these results show that the CNN model retained good predictive capability when little to no information for a specific subject was input to the CNN.

## Discussion

In this work, a Convolutional Neural Network was built and trained for automated detection of post-irradiation biochemical changes in Raman spectra of human breast cancer xenografts. The CNN discriminated irradiated versus nonirradiated tissue spectra acquired at an early timepoint following treatment and at clinically relevant doses with high classification accuracy, sensitivity, and specificity.

The CNN effectively classified irradiated and nonirradiated breast tissue spectra, with testing accuracies 94.6% and 92.1% for data collected 3 days post-irradiation, and 85.0% at day 1 post-irradiation. In addition, the model achieved significantly higher classification performance than the GBR-NMF-RF model for tissues harvested at day 3 post-irradiation. This finding agrees with other authors that report improvement in accuracy metrics of CNNs over common machine learning models for spectral analysis^24,25,27^. However, for spectra collected at day 1 post-irradiation, the CNN did not offer a major improvement in the classification results over GBR-NMF-RF. This could be due to the 1 day timepoint being too early for significant post-irradiation molecular changes to be identified in the spectra, or because the CNN hyper-parameters were optimized using only the data acquired at day 3 post-irradiation. Further hyper-parameter tuning of the CNN including the day 1 data set could potentially improve the results, but more work is required to test this hypothesis.

The classification of irradiated versus nonirradiated samples is not the final goal of the CNN. However, these initial results demonstrate that a one-dimensional CNN architecture is suitable for identifying discriminative patterns in tumour tissue Raman spectra and distinguishing different treatment groups at an early timepoint post-irradiation, with high accuracy and without the need for manual feature extraction (e.g., dimensionality reduction). In future work, the CNN can potentially be applied to distinguish Raman spectra of responding from nonresponding tumours to radiation therapy.

The subject-wise validation scheme was used to further test the CNN generalization ability when no information of a specific individual (e.g., mouse, patient) is given to train the model. This is the case in a clinical environment, where the model would be applied to make predictions on new patients, whose response to treatment is unknown, based on features learnt from a dataset of training patients. When all the data for a specific Raman map, tumour section, or mouse was held out for testing, there was variability in the percentage of correctly classified spectra among test subjects. This could be attributed to inter-mouse variability in radiation-induced spectral profiles or heterogeneity in the tumour dose distribution among different mapped regions. However, in agreement with the previous results, the CNN yielded higher percentage of correctly classified spectra for the majority of subjects than the GBR-NMF-RF framework, with average accuracies for test maps, sections, and individual mice 92.7%, 91.4%, and 83.7%, respectively.

Identifying individuals with radioresistant tumours before or early during treatment could help customize treatment for nonresponding patients and lead to improved therapeutic outcomes. Raman Spectroscopy (RS) offers the potential for identifying and monitoring radiation-induced biomolecular changes and signatures of radiation resistance in tumour cells and tissues. Convolutional Neural Networks are a state-of-the art deep learning tool for computer vision that perform efficient, automated feature extraction directly from data in an end-to-end learning manner, with outstanding classification performance. Hence, our group is interested in developing a Raman and CNN based predictive framework for rapid, automated, early characterization of tumour response to radiotherapy based on their degree of radiosensitivity or radioresistance.

In conclusion, the CNN can detect biochemical spectral changes in tumour tissue at an early time point following irradiation, without the need for previous manual feature extraction. A critical aspect in understanding the biological paths related to tumour radiation response and identify specific therapeutic targets, is to visualize the most critical spectral features/peaks extracted by the CNN to make its predictions. An example of explainable CNN models for spectral analysis was proposed by Zhang et al.^34^, wherein the authors implemented the Class Activation Map (CAM) technique to localize class-specific critical spectral peaks extracted by the CNN model in the classification of mid-infrared spectra of different strains of bacteria. Thus, future work will focus on developing methods to identify biochemical spectral signatures of radiation response captured by the CNN. The spectral features could then be assigned to specific biochemicals associated with radiation resistance or sensitivity. Finally, this initial study lays the foundation for developing a deep learning-based framework for characterization of tumour tissue responses based on their sensitivity to radiation treatment.

## Supplementary Information

Supplementary Information.

## Data Availability

All code and data generated and analysed during the current study are available from the corresponding author on reasonable request.
