# Supplementary material for: Raman spectroscopy and convolutional neural networks for monitoring biochemical radiation response in breast tumour xenografts
Source: Sci Rep. 2023 Jan 27;13:1530. doi: 10.1038/s41598-023-28479-2 (PMC9883395; doi:10.1038/s41598-023-28479-2)
Supplement: Supplementary file 1 — Supplementary Information. [file 41598_2023_28479_MOESM1_ESM.pdf]

Supplementary Material-Raman Spectroscopy and Convolutional Neural Networks for Monitoring Biochemical Radiation Response in Breast Tumour Xenografts

Alejandra M. Fuentes[1], Apurva Narayan[2,3], Kirsty Milligan[1], Julian J. Lum[4], Alex G. Brolo[5], Jeffrey L. Andrews[6], and Andrew Jirasek[1,*]

[1]Department of Physics, The University of British Columbia Okanagan Campus, Kelowna, Canada
[2]Department of Computer Science, Western University, London, Canada
[3]Department of Computer Science, The University of British Columbia Okanagan Campus, Kelowna, Canada
[4]Department of Biochemistry and Microbiology, The University of Victoria, Victoria, Canada
[5]Department of Chemistry, The University of Victoria, Victoria, Canada
[6]Department of Statistics, The University of British Columbia Okanagan Campus, Kelowna, Canada
[*]andrew.jirasek@ubc.ca

Table S1. Raman biochemical library for nonnegative matrix factorization

Name	Group
Alanine	Amino acid
Arginine	Amino acid
Asparagine	Amino acid
Citric acid	TCA cycle
CoEnzymeA	TCA cycle
Cysteine	Amino acid
DNA	Nucleic acid
Glucose	Carbohydrate
Glutamic acid	Amino acid
Glutathione	Antioxidant
Glycerol	Sugar alcohol
Glyceryl tripalmitoleate	Triglyceride
Glycogen	Carbohydrate
Histidine	Amino acid
Isoleucine	Amino acid
Lactose	Carbohydrate
Mannose	Carbohydrate
Methionine	Amino acid
Oleic acid	Fatty acid
Palmitic acid	Fatty acid
Phenylalanine	Amino acid
Phosphatidylcholine	Phospholipid
Phosphatidylserine	Phospholipid
Phosphatidylinnositol	Phospholipid
Serine	Amino acid
Stearic acid	Fatty acid
Triglycerides	Lipid
Tryptophan	Amino acid
Tyrosine	Amino acid
Valine	Amino acid
Collagen	Protein

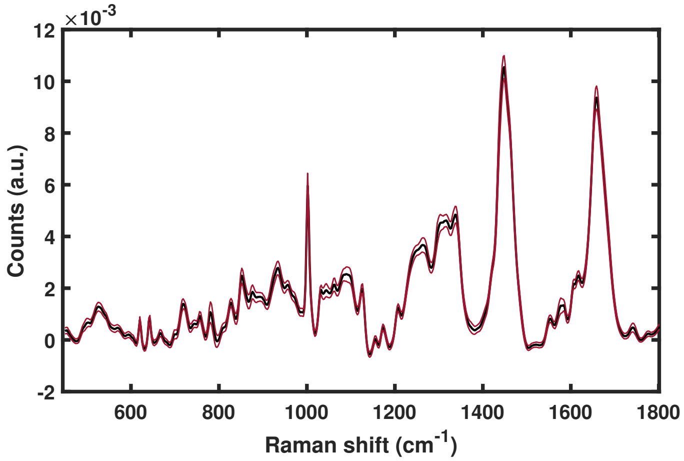

Figure S1. Mean Raman spectrum (black) of breast cancer xenografts exposed to 0 and 15 Gy and acquired at day 1 post-irradiation. The standard deviation at each wavenumber is shown in red.

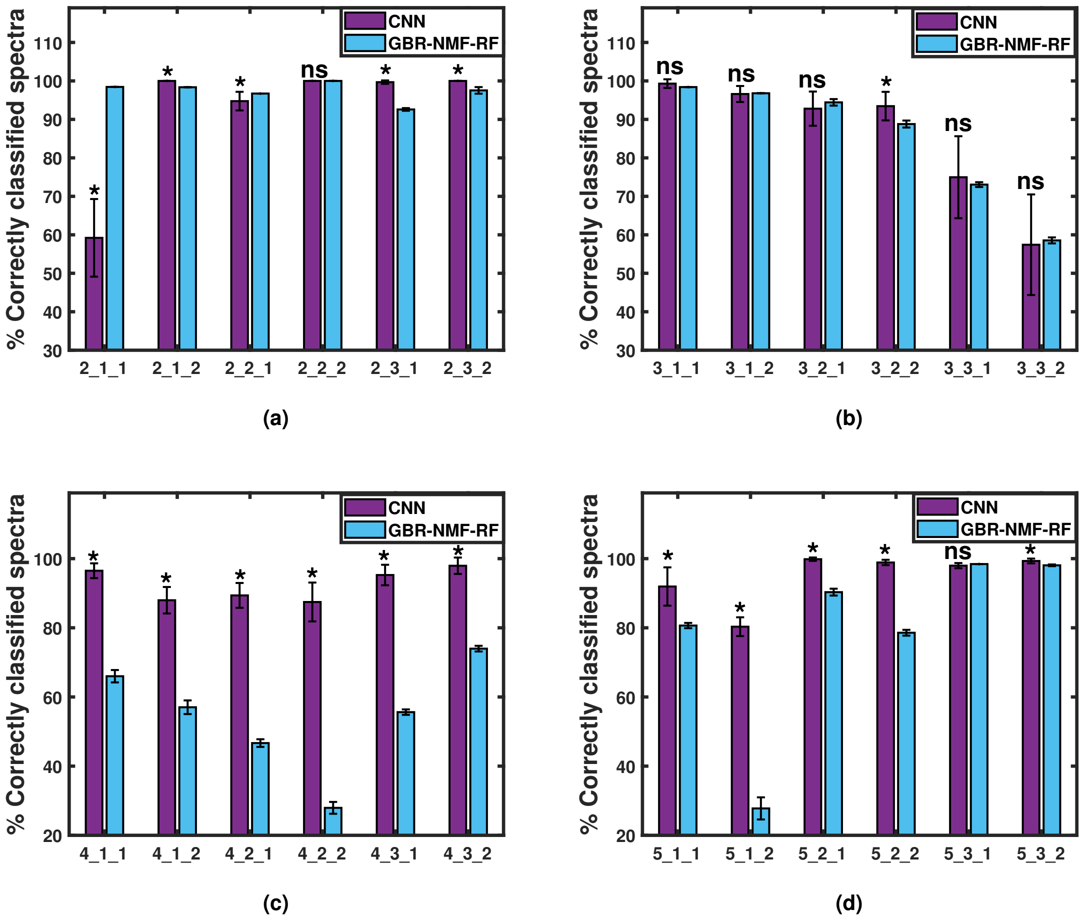

Figure S2. Percentage of correctly classified spectra (test accuracy) corresponding to each tumour Raman map removed from the training set (leave-one-map-out validation). Map labels given as mouse number_(section #)_(map #).* Represent significant difference between CNN and GBR-NMF-RF ($p<0.05$), ns= not significant.

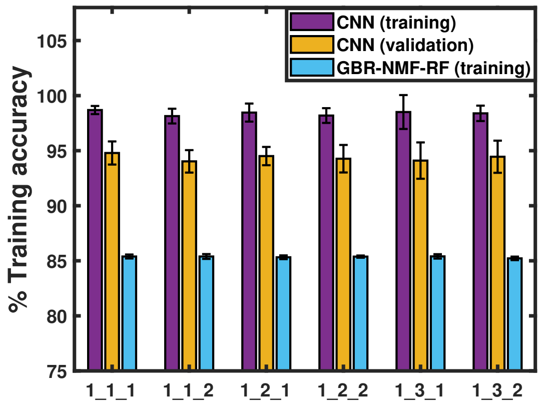

Figure S3. Training accuracy corresponding to each Raman map of mouse 1 removed from the training set (leave-one-map-out validation) for CNN (violet) and GBR-NMF-RF (blue), and validation accuracy for CNN (orange). Map labels given as mouse number_(section #)_(map #).

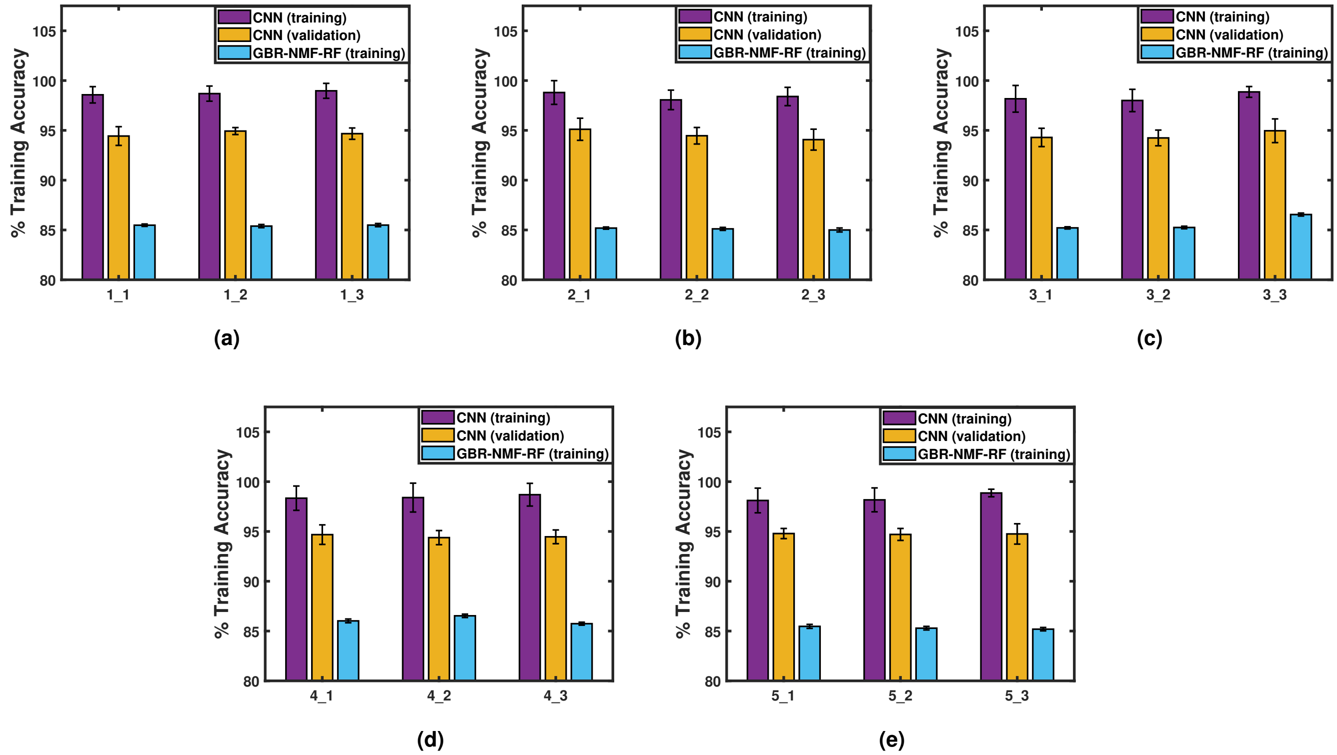

Figure S4. Training accuracies corresponding to each tumour section removed from the training set (leave-one-section-out validation) for CNN (violet) and GBR-NMF-RF (blue), and validation accuracy for the CNN model (orange). Section labels given as mouse number_(section #).
